# 384. National Trends and Racial Disparities in Sepsis-Related Mortality in Children in the United States, 1999-2019

**DOI:** 10.1093/ofid/ofae631.119

**Published:** 2025-01-29

**Authors:** Ladonna Boasiako, Patricia pappoe, Manye Ami Asante-Darko, Felix Nii Saka Allotey, Jennifer Peseo, Chidinma S Ononogbu, Fredrick Dapaah-Siakwan

**Affiliations:** No Affiliation, Brandywine, Maryland; Unaffiliated, Tucson, Arizona; Department of Child Health, Korle Bu Teaching Hospital, Accra, Greater Accra, Ghana; Ho Teaching Hospital, Ho, Volta, Ghana; University of Ghana Medical Centre, Accra, Greater Accra, Ghana; Korle Bu Teaching Hospital, Accra, Accra, Greater Accra, Ghana; Valley Children's Pediatric Residency Program, Madera, California

## Abstract

**Background:**

There is conflicting data on the racial differences in sepsis-related mortality rate (SRMR) in children and little is known about the trends in SRMR in children in the United States (US). We examined the national trends and racial disparities in SRMR in children in the US from 1999 to 2019.

Sepsis-related mortality rate in US children stratified by age, gender, race, and geographic region.

**Figure:**
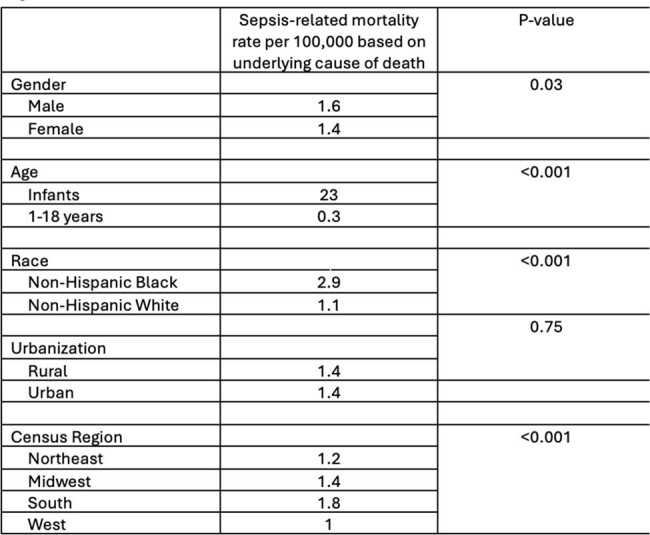

Sepsis-related mortality rate in US children stratified by age, gender, race, and geographic region.

**Methods:**

This was a retrospective cross-sectional analysis of national death certificate data from the CDC WONDER database from 1999-2019. We used ICD-10 codes to identify children aged 0-18 years who had bacterial sepsis as the underlying cause of death. We excluded deaths from viral, fungal, parasitic, tuberculous, and rickettsial illnesses. The crude SRMR was calculated per 100,000 population and further stratified by age, gender, race, and census region. Racial disparity was evaluated by the NHB-to-NHW SRMR ratio. The Mann-Whitney U test was used to compare two groups and *P* < 0 .05 defined statistical significance. We evaluated temporal trends with Joinpoint regression, expressed as average annual percentage change (AAPC) with 95% confidence intervals (CI). Next, we repeated the analysis with any mention of bacterial sepsis as a cause of death.

**Figure 1.**
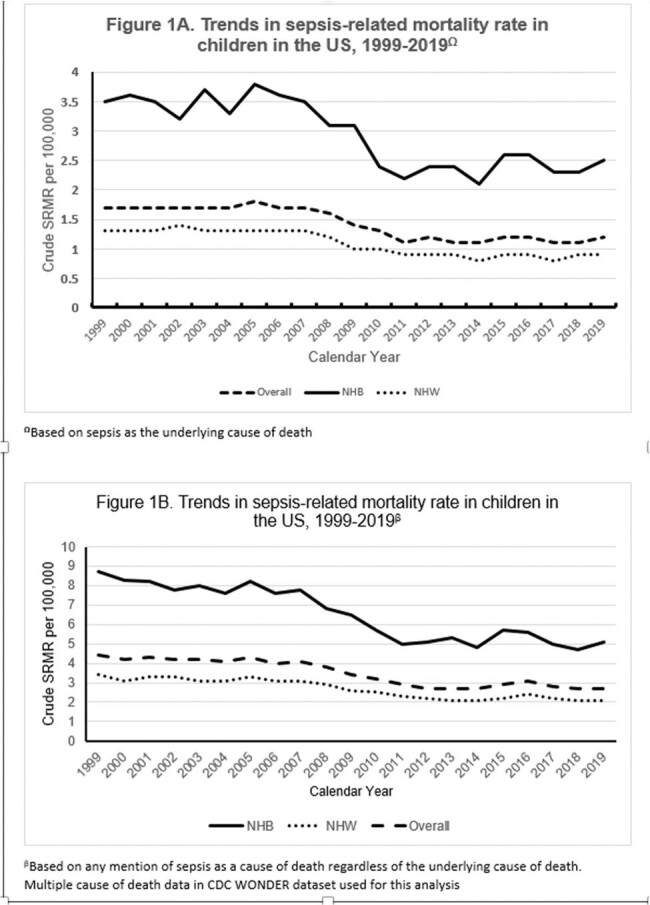
Trends in sepsis-related mortality rate in children in the US, 1999-2019.

**Results:**

Among 1.6 billion children, 23,466 had bacterial sepsis as the underlying cause of death [1.4 per 100,000; CI, 1.4 -1.5)]. The SRMR differed by age, gender, race, and geographic region (Table 1). The overall SRMR decreased from 1.7 in 1999 to 1.2 in 2019 (AAPC -1.9%; CI: -2.4, -1.3%) [Figure 1]. The SRMR decreased in males (1.9 to 1.2; AAPC -2.2%, CI: -2.5, -1.9), females (AAPC -2%, CI: -2.5, -1.6), NHB (AAPC -1.7%, CI: -2.3, -0.9); NHW (AAPC -2.1%, CI: -2.4, -1.6), rural (AAPC -2.5%, CI: -3.4, -1.5) and urban areas (AAPC -1.9%, CI: -2.3, -1.5), and in all census regions. The overall NHB-to-NHW SRMR ratio was 2.7 and there was no significant change during the study period. When limited to any mention of sepsis as the cause of death, the differences and trends in SRMR remained unchanged (Figure 1B).

**Conclusion:**

The SRMR declined significantly overall and for all demographic groups. The persistent disparity in SRMR between NHB and NHW suggests differential access to care, recognition and prompt treatment of sepsis. Future studies should unravel the underlying causes of this disparity in mortality outcomes.

**Disclosures:**

**All Authors**: No reported disclosures

